# Wnt4 Participates in the Formation of Vertebrate Neuromuscular Junction

**DOI:** 10.1371/journal.pone.0029976

**Published:** 2012-01-12

**Authors:** Laure Strochlic, Julien Falk, Evelyne Goillot, Séverine Sigoillot, Francine Bourgeois, Perrine Delers, Jérôme Rouvière, Amanda Swain, Valérie Castellani, Laurent Schaeffer, Claire Legay

**Affiliations:** 1 Université Paris Descartes, Sorbonne Paris Cité, France; 2 Université Lyon, Villeurbanne, France; 3 Université de Lyon, Lyon, France; 4 Section of Gene Function and Regulation, Institute of Cancer Research, London, United Kingdom; University of Colorado, Boulder, United States of America

## Abstract

Neuromuscular junction (NMJ) formation requires the highly coordinated communication of several reciprocal signaling processes between motoneurons and their muscle targets. Identification of the early, spatially restricted cues in target recognition at the NMJ is still poorly documented, especially in mammals. Wnt signaling is one of the key pathways regulating synaptic connectivity. Here, we report that Wnt4 contributes to the formation of vertebrate NMJ *in vivo*. Results from a microarray screen and quantitative RT-PCR demonstrate that Wnt4 expression is regulated during muscle cell differentiation *in vitro* and muscle development *in vivo*, being highly expressed when the first synaptic contacts are formed and subsequently downregulated. Analysis of the mouse Wnt4−/− NMJ phenotype reveals profound innervation defects including motor axons overgrowing and bypassing AChR aggregates with 30% of AChR clusters being unapposed by nerve terminals. In addition, loss of Wnt4 function results in a 35% decrease of the number of prepatterned AChR clusters while Wnt4 overexpression in cultured myotubes increases the number of AChR clusters demonstrating that Wnt4 directly affects postsynaptic differentiation. In contrast, muscle structure and the localization of several synaptic proteins including acetylcholinesterase, MuSK and rapsyn are not perturbed in the Wnt4 mutant. Finally, we identify MuSK as a Wnt4 receptor. Wnt4 not only interacts with MuSK ectodomain but also mediates MuSK activation. Taken together our data reveal a new role for Wnt4 in mammalian NMJ formation that could be mediated by MuSK, a key receptor in synaptogenesis.

## Introduction

Neuromuscular junctions (NMJ) form in three steps that include specific nerve-muscle recognition, synaptic differentiation, and maturation of the synapse. Early during development, before innervation takes place, muscles are “prepatterned” and acetylcholine receptors (AChRs) are found localized in a central band of the muscle [1], [2]. This muscle cell autonomous process is believed to instruct the navigating motor growth cones on their way toward their appropriate target field within the muscle [3], [4]. To date, several molecules have been identified as key to this early synaptic targeting including the muscle specific kinase (MuSK), a tyrosine kinase receptor known to play a central role in the formation of NMJs and the low density lipoprotein receptor-related protein 4 (LRP4) [4], [5]. Innervation provides at least two neural secreted factors agrin and acetylcholine (ACh) that reshape the size and the distribution of prepatterned AChR clusters [6]. Agrin binds the LRP4/MuSK complex, to activate MuSK [7], [8]. Activated MuSK in turn induces signaling pathways leading to the clustering of synaptic proteins in the postsynaptic membrane [9]. At the same time, agrin counteracts ACh-elicited dispersal of AChR clusters resulting in the removal of aneural AChR clusters and stabilization of nerve associated AChRs clusters [10], [11].

Wnts are secreted glycoproteins that regulate key aspects of neuronal development including axon guidance and synaptic differentiation [12], [13], [14], [15]. These processes are mediated by a variety of Wnt signal transduction pathways generated by a wide range of Wnts proteins and cognate receptors (the Wnt homepage: www.stanford.edu/~rnusse/wntwindow). The main class of Wnt receptors are the seven-pass transmembrane Frizzled (Fz) receptors. In addition, two tyrosine kinase receptors, Ryk/Derailed and ROR have been reported as non conventional Wnt receptors [13]. Interestingly, MuSK contains within its extracellular domain a Fz-like, cysteine-rich domain (CRD) homologous to the domain on Fz receptors to which Wnt binds [16]. In *Drosophila*, the Wnt homologue Wingless (Wg) is required for NMJ maturation and coordinated development of pre- and postsynaptic structures [17]. In *C.elegans* however, lin44, the Wnt homologue acts as a repulsive cue inhibiting premature synapse formation [18]. In vertebrates, 19 Wnt proteins have been reported and emerging data indicate that several regulate disparate events in NMJ formation. For example, Wnt3 and Wnt3a affect AChR clustering [19], [20]. Also, several intermediates in the Wnt pathway such as Dishevelled (Dvl), beta-catenin, APC, GSK-3 and Casein kinase 2 are accumulated at the NMJ and mutants for some of these molecules display abnormal AChR clustering [9], [21], [22]. Interestingly, Granato and co-workers have shown in zebrafish that Wnt11r interacts with the *unplugged*/MuSK CRD ectodomain and restricts innervation to the central zone of the muscle [3].

Our studies provide compelling evidence that Wnt4 is involved in the formation of mammalian NMJs. We show that Wnt4 level of expression is highly expressed in the early steps of NMJ development when the first synaptic contacts are formed *in vivo* and is subsequently downregulated. Analysis of the NMJ phenotype of the Wnt4−/− mice embryos revealed profound innervation defects: 1) overgrowth of primary branches across the muscle that bypassed AChR aggregates 2) increase size of aggregates with significantly greater AChR molecules 3) increase in the width of the endplate band of clusters and a significant number (30%) of uninnervated AChR clusters. In contrast, the localization of several key components of the synapse including acetylcholinesterase (AChE), MuSK and Rapsyn is not perturbed in the Wnt4 mutant. Also, we show that loss of Wnt4 function results in a decrease (35%) of prepatterned AChR, while in contrast, Wnt4 enhances AChR clustering in cultured myotubes demonstrating that Wnt4 directly affects postsynaptic differentiation. Finally, we report that Wnt4 interacts with MuSK via its CRD domain, this interaction leading to MuSK activation through tyrosine phosphorylation. Together, these data reveal that Wnt4 is a new player in the formation of mammalian NMJs.

## Results

### Wnt4 expression during neuromuscular junction development

**Figure 1:**
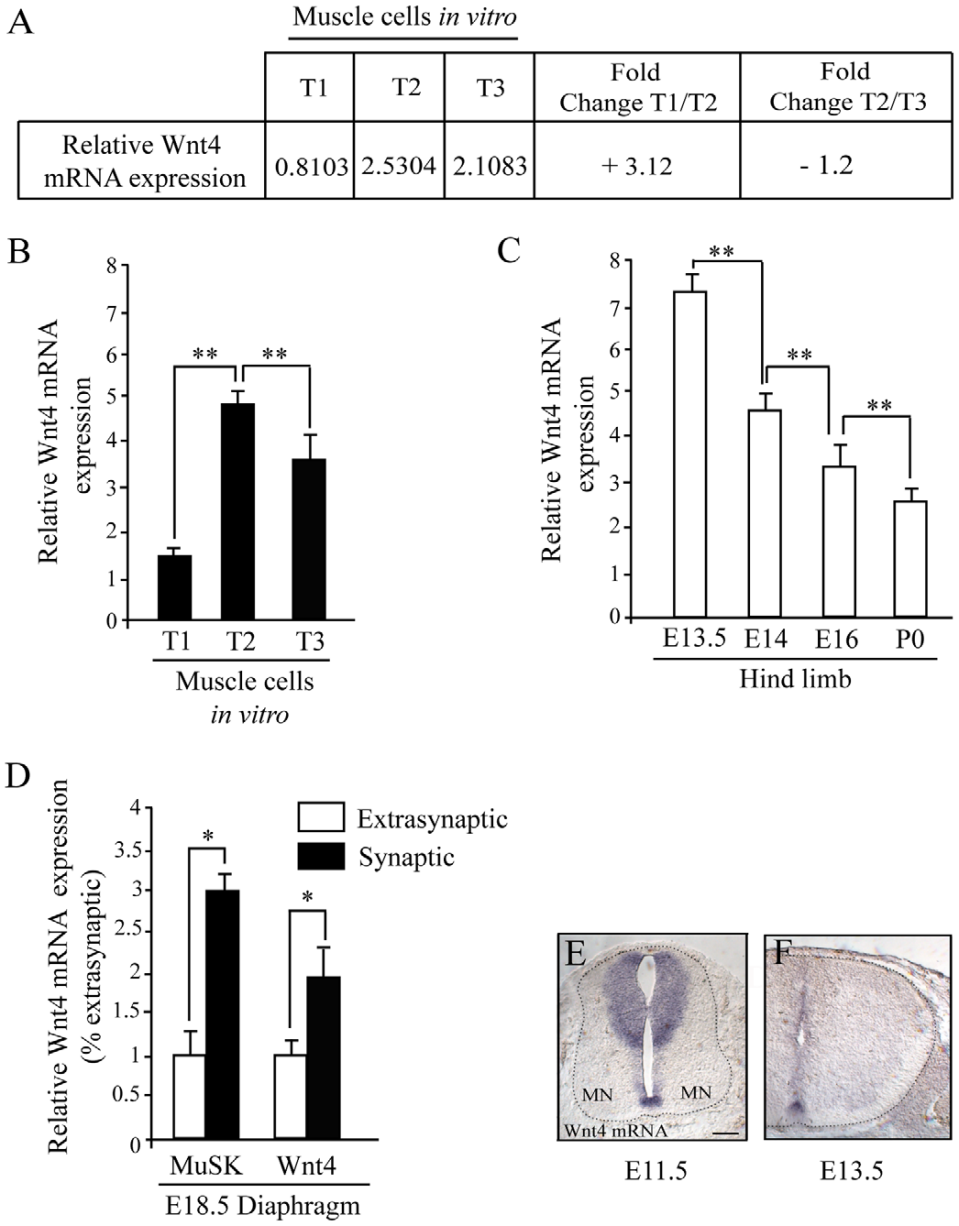
Wnt4 expression during neuromuscular junction development. (**A**) Table showing results of Affymetrix microarrays data comparing relative Wnt4 mRNA expression during myotube differentiation, between stages T1/T2 and T2/T3 (see Materials and Methods). Relative Wnt4 mRNA is upregulated more than three fold between stage T1/T2 and downregulated more than one fold between stages T2/T3. (**B and C**) Real time RT-PCR quantification of relative Wnt4 mRNA expression during myotube differentiation (B, stages T1, T2 and T3) and hind limb development (C, embryonic stages E13.5, E14, E16 and newborn mice P0, N = 6 embryos tested for each stage). Relative Wnt4 mRNA expression is significantly increased between stages T1/T2 and further downregulated between stages T2/T3 and decreases as the limb developed. (**D**) Real time RT-PCR quantification of relative MuSK and Wnt4 mRNA expression in synaptic and extrasynaptic regions of diaphragms from stage E18.5 embryos. Relative MuSK and Wnt4 expression are three and two fold increased in synaptic compared to extrasynaptic regions respectively. Results are represented as relative expression (2^−Δ*C*t^ versus reference gene ×100, N = 3). (**E and F**) In situ hybridization with probes for Wnt4 mRNAs in E11.5 and E13.5 spinal cord sections (thoracic level) of wild type mice embryos (N = 3 embryos tested for each condition). Wnt4 mRNA is expressed in the floor plate and dorsal spinal cord but not in motoneurons (MN). Error bars show means ± SEM from three independent experiments. *P<0.05; **P<0.001; Mann-Whitney *U* test. Scale bar: in E, 20 µm for E and F.

In a large screen aiming at exploring the expression profiles of mRNAs during muscle differentiation, we performed a microarray analysis at three different muscle cell stages. The muscle cell line and the stages used (T1, T2 and T3) have been previously described [23]. Briefly, the transition T1 to T2 can be correlated *in vivo* to the stage at which muscle begins to be innervated, a process that takes place in mice between E13.5 and E14 just after myoblast fusion into myotubes. The transition T2 to T3 corresponds to further maturation of muscle cell marked by the appearance of muscle cell contraction. Results from the microarray and quantitative RT-PCR experiments revealed that Wnt4 mRNA levels were upregulated at T2 compared to T1 (3-fold) and then downregulated as muscle differentiation proceeded between T2 and T3 (Fig. 1A and B). These results suggest that Wnt4 is expressed by myotubes when the postsynaptic compartment differentiates *in vitro*.

Consistent with our *in vitro* data, Wnt4 mRNA pattern of expression was also regulated *in vivo* during hind limb development (stages E13.5; E14; E16 and P0). Indeed, Wnt4 mRNA was already expressed at stage E13.5 when NMJs start to form and decreased as limb development progress (Fig. 1C). Given that non-muscle tissus in the limb have been reported to express Wnt4, we performed RT-PCR experiments on dissected diaphragm muscles [24]. Equivalent size tissue samples of synapse-rich and extrasynaptic sites from stage E18.5 wild type diaphragms were microdissected, labeled with α-bungarotoxin (BGT) and MuSK and Wnt4 gene expression in these two regions were compared. Relative MuSK mRNA expression, used as a positive control for synapse enrichment, was three fold increased in synaptic-rich compared to extrasynaptic regions (Fig. 1D). Relative Wnt4 mRNA expression was detected in diaphragm muscles and found to be two fold enriched in synaptic regions indicating that Wnt4 mRNA is expressed by muscle and patterned as other key regulators of synaptogenesis including MuSK (Fig. 1D).

Finally, we asked whether developing motoneurons express Wnt4. In situ hybridization performed on spinal cord sections from stages E11.5 and E13.5 wild type mice embryos showed that Wnt4 mRNA is expressed in the floor plate and in the dorsal zone as previously described [25]. However, no signal was detected in the ventral zone where motoneurons are located (Fig. 1E and F). We conclude that Wnt4, highly expressed in muscles when the first synaptic contacts are formed *in vivo* is likely to play a role in synapse formation.

### Aberrant neuromuscular junction innervation in muscles of Wnt4−/− embryos

**Figure 2:**
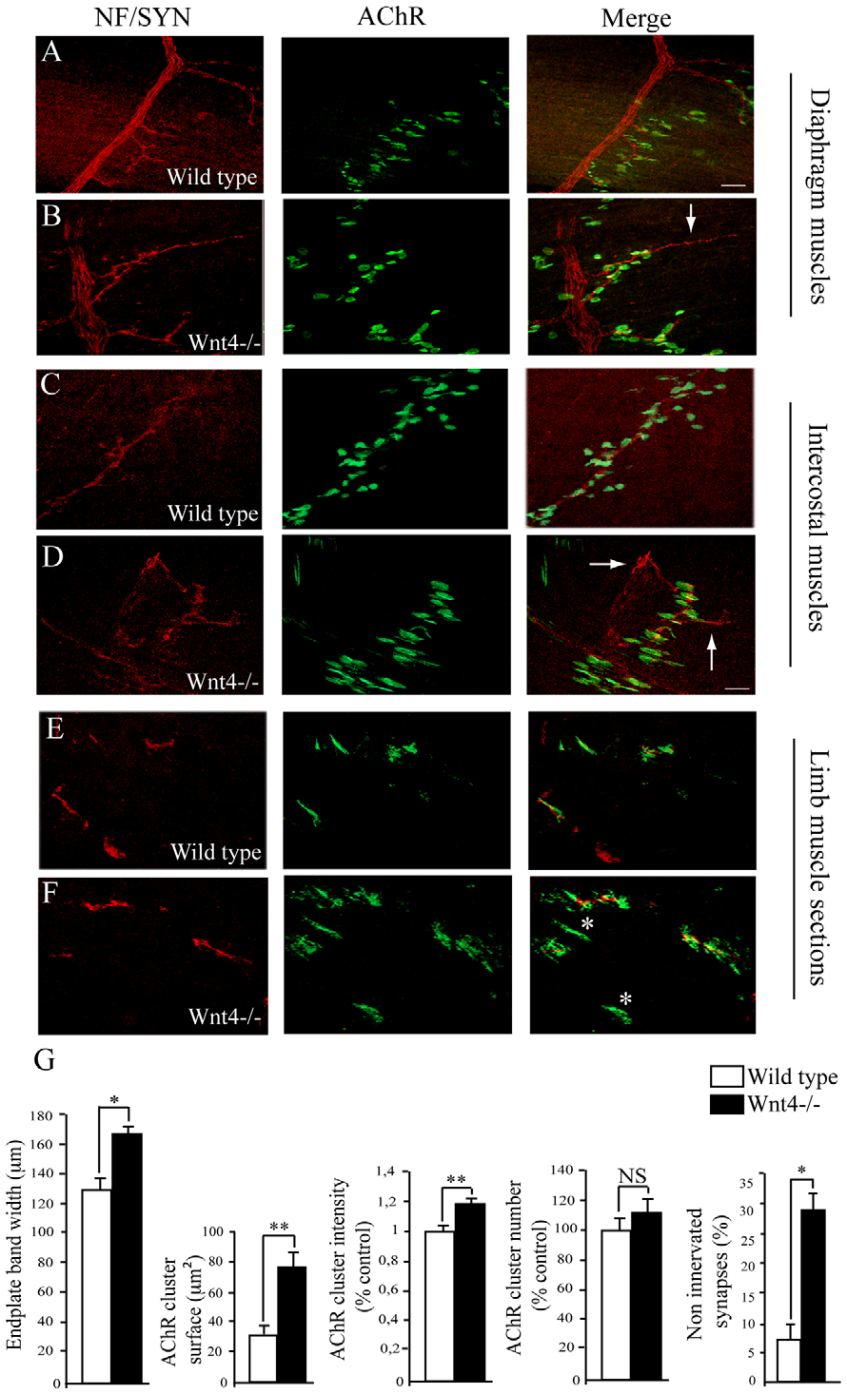
Aberrant neuromuscular junction innervation in muscles of Wnt4−/− embryos. (**A–F**) Confocal images of whole mount diaphragm (A and B), intercostal muscles (C and D) or cross sections of hind limb muscles (E and F) from stage E18.5 control littermates (wild type, A, C and E) or Wnt4−/− embryos (B, D and F) stained with neurofilament (NF, red) and synaptophysin (Syn, red) antibodies together with α-bungarotoxin (AChRs, green). Examples of nerve terminals passing through and projecting beyond the central band of AChR clusters in mutant diaphragm or intercostal muscles are indicated by white arrows in the merged image in B and D. Examples of non innervated synapses in mutant limb muscles are indicated by white stars in the merged image in F. (**G**) Measurement of AChR endplate band width, AChR clusters surface, α-bungarotoxin fluorescence signal intensity (numbers of AChR clusters tested: 95 in control and 76 in Wnt4−/−), AChR cluser number and number of non innervated synapses (%) in limb muscle cross sections (numbers of synapses counted: 35 in control and 28 in Wnt4−/−; N = 3 for Wnt4 mutants and N = 4 for control littermates embryos). Error bars show means ± SEM. *P<0.05; **P<0.001; Mann-Whitney *U* test. NS, non significant. Scale bars: in the merged image in A, 60 µm for A and B; in the merged image in D, 30 µm for C, D, E and F.

To test the role of Wnt4 in NMJ innervation, we analyzed NMJ formation in three different muscle types (diaphragm, intercostal and limb muscles) from E18.5 Wnt4−/− mutant embryos or control littermates. Wnt4 mutant mice die within 24 h after birth due to a defect in kidney formation [26]. Whole mount diaphragm or intercostal muscle preparations were stained with neurofilament (NF) and synaptophysin (Syn) together with α-BGT to visualize nerves and AChR clusters (Fig. 2A, B, C, D). In control embryos, motor axon projections terminated close to the main nerve trunk and nerve terminals juxtaposed to AChR clusters as previously described [27]. In Wnt4−/− embryos, however, the main nerve trunk appeared defasciculated or overbranching. Nevertheless it ran in the center of the muscle indicating that the positioning of the nerve is rather normal but not the bundling of axons within the nerve (Fig. 2B and D). Moreover, axons extending from the nerve trunk passed through and projected beyond the central band of AChR clusters (Fig. 2B and D, white arrows). Analysis of intercostal muscles revealed a change in the distribution of AChR clusters in Wnt4 mutants (Fig. 2G). The width of the central band of clusters was significantly broader (≈120 µm in wild type vs. ≈160 µm in Wnt4 mutants; Fig. 2G). In addition, AChR clusters area was also significantly increased (≈30 µm^2^ in wild type vs. ≈75 µm^2^ in Wnt4 mutants; Fig. 2G) and we found a 20% increase in the α-BGT signal intensity compared to wild type embryos (Fig. 2G). No difference in the number of AChR clusters was observed in the Wnt4 mutant compared to control (Fig. 2G).

Normally, most of the uninnervated AChR clusters disassemble so that only few are not apposed to nerve terminals. Surprisingly, we found that in the Wnt4−/− mutant embryos, ≈30% of AChR clusters were not apposed to nerve terminals compared to ≈7% in wild type embryos, a more than 4 fold difference in vacant postsynaptic densities (Fig. 2E, F, G).

### Synaptic markers are localized at the NMJ in Wnt4−/− embryos

**Figure 3:**
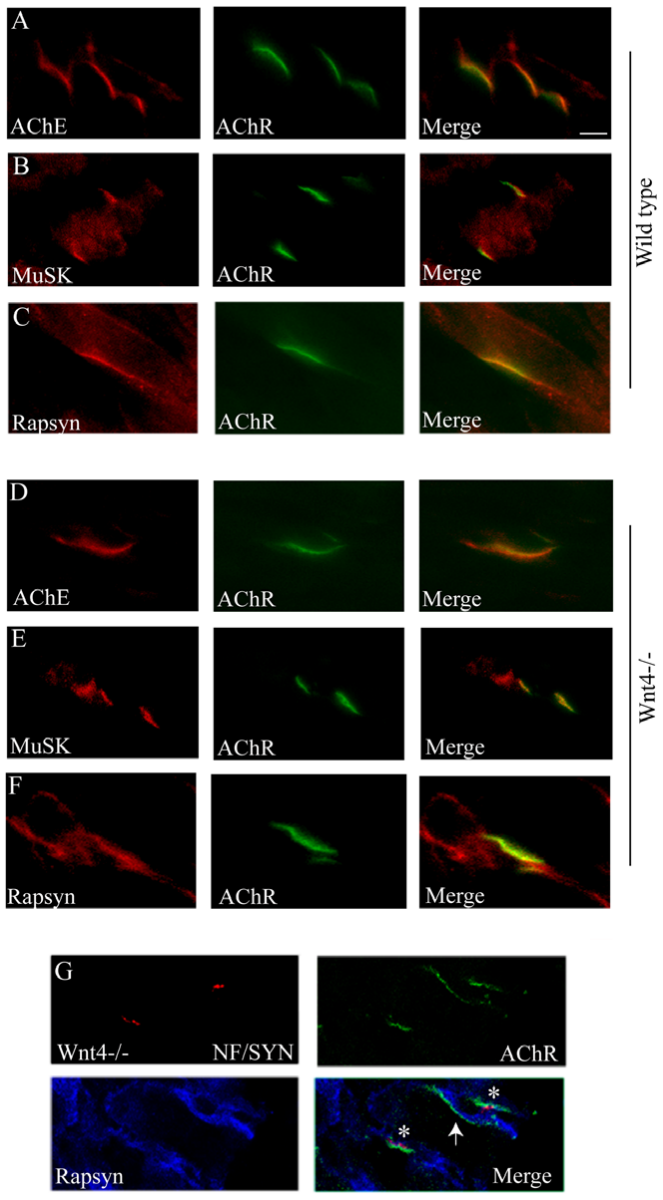
Synaptic markers are localized at the NMJ in Wnt4−/− embryos. (**A–F**) Hind limb muscle cross sections from stage E18.5 control littermates (A–C) or Wnt4−/− embryos (D–F) stained with AChE (red, A and D), MuSK (red, B and E) or rapsyn (red, C and F) antibodies together with α-bungarotoxin (AChR, green). AChE, MuSK and rapsyn colocalized with AChR at the NMJ of wild type and Wnt4−/− mutant embryos (15 cross sections from 2 Wnt4 mutants and control littermates were analyzed for each condition). (**G**) Confocal images of hind limb muscles cross sections from stage E18.5 Wnt4−/− embryos stained with neurofilament (NF, red), synaptophysin (Syn, red) and rapsyn (blue) antibodies together with α-bungarotoxin (AChRs, green). Examples of innervated and non innervated synapses are indicated by white stars and arrowhead respectively. Non innervated synapses still expressed the rapsyn protein (15 cross sections from 2 for Wnt4 mutants and control littermate embryos). Scale bar: in the merged image in A, 20 µm.

To further analyze the postsynaptic defects observed in the Wnt4 mutant, we next asked whether the localization of key synaptic proteins at Wnt4−/− NMJ is perturbed. Thus, we examined the distribution of several proteins known to be concentrated in the synaptic basal lamina, in the postsynaptic membrane or beneath the postsynaptic membrane of NMJs [6]. Limb muscle cross sections from stage E18.5 Wnt4−/− or control littermates were stained for acetylcholinesterase (AChE), MuSK or rapsyn together with α-BGT (Fig. 3A, B, C, D, E, F). No obvious differences could be detected in the localization of these proteins at the mutant NMJs compared to control. Since 30% of Wnt4−/− AChR clusters were not innervated (Fig. 2G), we then wondered if these non innervated Wnt4 deficient AChR clusters still contained the expected complement of postsynaptic proteins. Limb muscle cross sections from stage E18.5 Wnt4−/− were stained with rapsyn and NF/Syn together with α-BGT (Fig. 3G). Rapsyn was detected both in innervated and non innervated AChR clusters and overlapped AChR clusters indicating that the proper synaptic localization of at least rapsyn is not affected in Wnt4−/− mutant embryos.

### Wnt4 affects muscle prepatterning and AChR clustering in muscle cells

**Figure 4:**
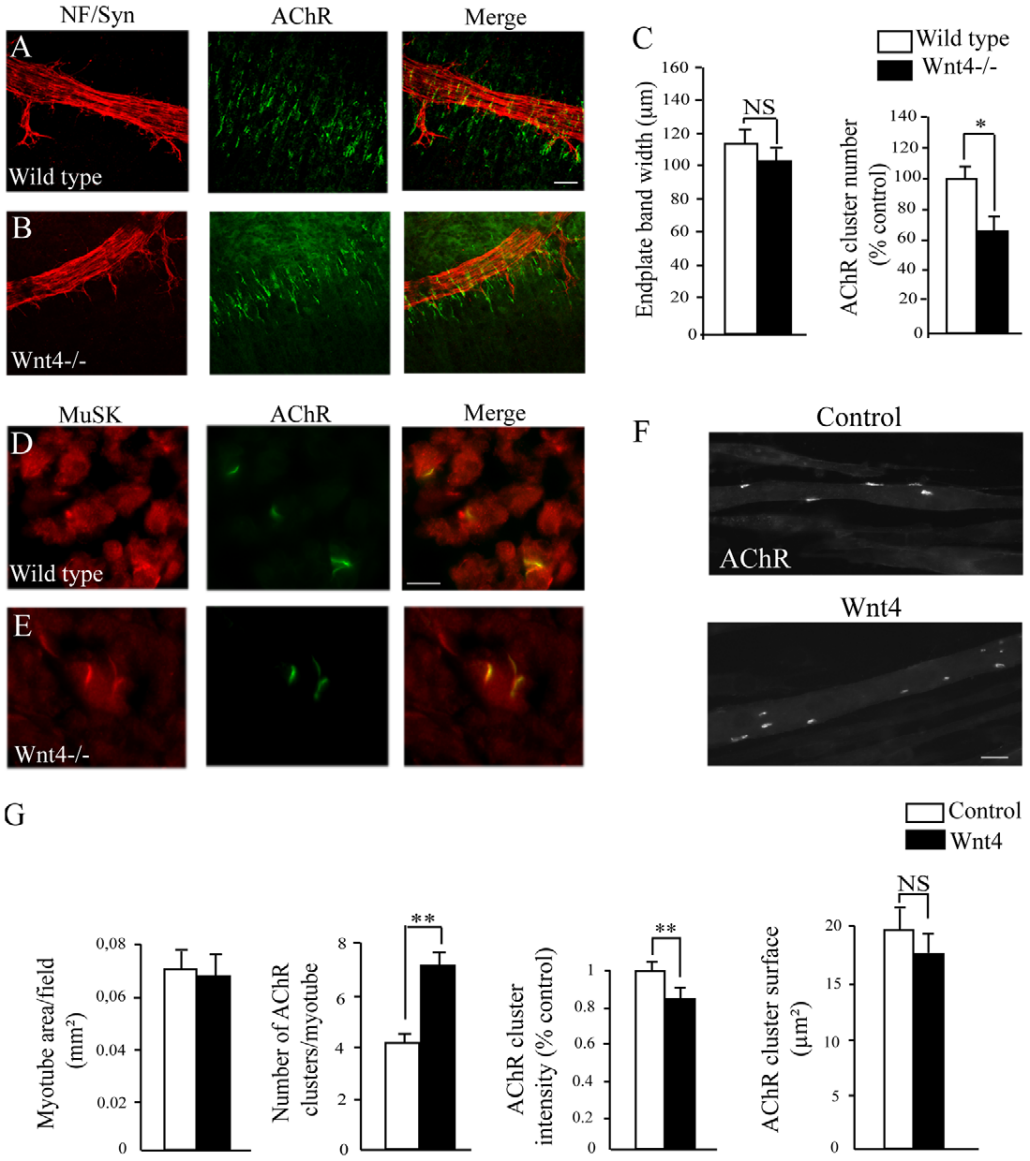
Wnt4 affects muscle prepatterning and AChR clustering in muscle cells. (**A and B**) Confocal images of whole mount intercostal muscles from stage E14 control littermates (wild type, A) or Wnt4−/− embryos (B) stained with neurofilament (NF, red) and synaptophysin (Syn, red) antibodies together with α-bungarotoxin (AChRs, green). Both in wild type and Wnt4−/− mutant embryos, AChR clusters were detected (N = 2 for Wnt4 mutants and N = 2 for control littermate embryos). (**C**) Quantification analysis of the AChR endplate band width and number of prepatterned AChR clusters. (**D and E**) Hind limb muscle cross sections from stage E14 control littermates (C) or Wnt4−/− embryos (D) stained with MuSK (red) antibodies together with α-bungarot oxin (AChR, green). MuSK colocalized with AChR at the NMJ of wild type and Wnt4−/− mutant embryos (10 cross sections from 2 Wnt4 mutants and control littermates were analyzed for each condition). (**F**) Examples of myotubes stained with α-bungarotoxin (AChR) upon control or Wnt4 treatment. (**G**) Measurements of the myotube area/field, the number of AChR clusters/myotube, the AChR cluster fluorescence signal intensity and the average AChR cluster area (50 AChR clusters for control and 65 for Wnt4 treated myotubes were analyzed). Wnt4 treatment induced an increase in the number of AChR clusters/myotube. However, AChR cluster fluorescence signal intensity was significantly reduced in Wnt4 treated myotubes. Error bars show means ± SEM. *P<0.05; **P<0.001; Mann-Whitney *U* test. NS, non significant. Scale bar: in A, 100 µm for A and B; in D, 30 µm for D and E; in F, 20 µm.

In order to understand the origin of the phenotype observed at E18.5, we wondered whether Wnt4 affects muscle prepatterning earlier during development. Interestingly, Wnt11 has been shown to control AChR prepatterning and axon guidance in Zebrafish [3]. Whole mount intercostal muscles from E14 Wnt4−/− embryos or control littermates were stained with α-BGT to visualize synaptic prepatterning just prior to innervation. In the Wnt4 mutant, the nerve trunk was localized in central region of the muscle and fasciculation appeared normal suggesting that the phenotype observed does not result from altered axon navigation or fasciculation (Fig. 4A and B). AChR clusters were detected in the central zone of the muscle in the Wnt4 mutant (Fig. 4A and B). Quantification analysis did not reveal any significant difference in the AChR endplate band width between Wnt4−/− mutants and control littermates (Fig. 4C). Interestingly, we found a 35% decrease in the number of prepatterned AChR clusters in Wnt4 mutant suggesting that Wnt4 regulates the number of prepatterned AChR clusters (Fig. 4C).

AChR clusters formation and maintenance critically depends on MuSK signaling [6]. Therefore, we examined MuSK localization during AChR prepatterning. In agreement with our results at E18.5, there was no obvious difference in MuSK localization at the NMJ of limb muscle cross sections from Wnt4−/− or control littermates where MuSK completely overlapped AChR clusters (Fig. 4D and E).

This result prompted us to test whether Wnt4 could influence directly AChR clustering in muscle cells in culture. T2 stage myotubes were treated with recombinant Wnt4 protein overnight and stained with α-BGT to visualize and quantify AChR clustering (Fig. 4F). No difference in the average myotube and AChR cluster size were observed following Wnt4 exposure. Importantly, in contrast to the lack of Wnt4 *in vivo* at E14, Wnt4 treatment enhanced the number of AChR clusters compared to control myotubes and we observed a decrease in the AChR fluorescence intensity upon Wnt4 treatment (Fig. 4G). These results show that Wnt4 directly affects AChR clustering and activates a muscle signaling pathway regulating postsynaptic differentiation prior to innervation.

### Wnt4 does not alter muscle structure but modify fiber type composition

**Figure 5:**
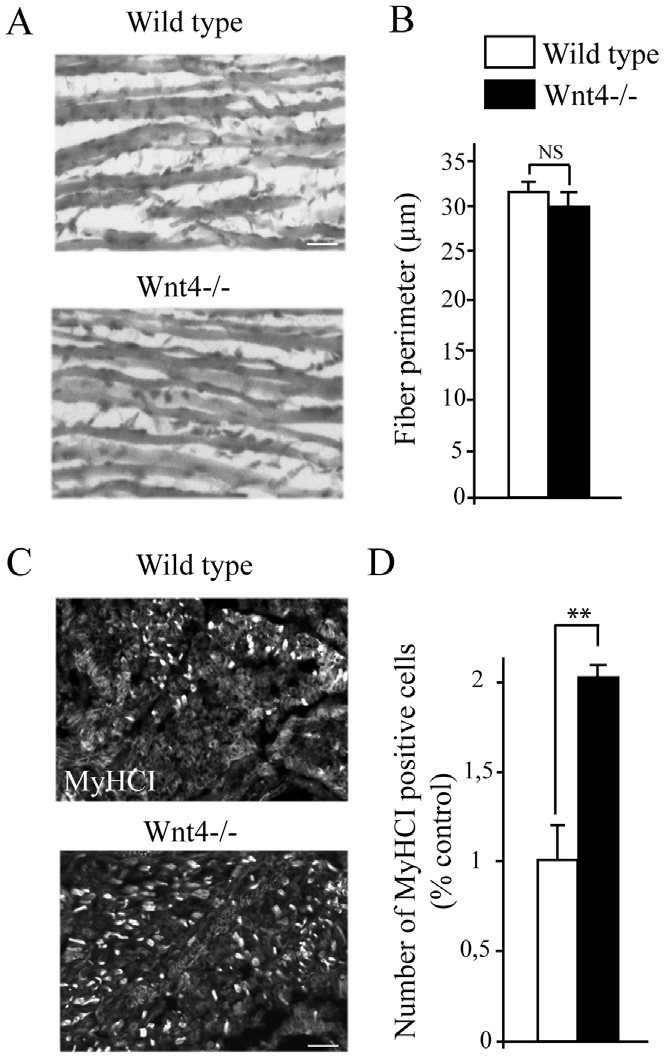
Wnt4 does not alter muscle structure but modifiy fiber type composition. (**A**) Histological analysis of hind limb muscle cross sections from stage E18.5 control littermate (wild type) or Wnt4−/− embryos stained with heamatoxylin/eosin. The muscle gross organization was not affected in the Wnt4−/− embryos (N = 3 for Wnt4 mutants and N = 4 for control littermate embryos). (**B**) Measurement of muscle fibers perimeter. No significant difference in limb muscle section perimeter from Wnt4−/− mutant compared to wild type was detected. (**C**) Hind limb muscle cross sections (soleus level) from stage E18.5 control littermates (wild type) or Wnt4−/− embryos stained with myosin heavy chain I (MyHCI) antibodies (N = 2 for Wnt4 mutants and control littermate embryos). (**D**) Measurement of the number of MyHCI postive cells. The number of MyHCI positive cells was increased in Wnt4−/− mutant compared to wild type. Error bars show means ± SEM. **P<0.001; Mann-Whitney *U* test. NS, non significant. Scale bars: in A, 30 µm; in C, 10 µm.

Since Wnt4 has been implicated in muscle differentiation and has been reported to influence muscle fibers type, we wondered whether the change in AChR clusters distribution and size observed at E18.5 could be the result of an impaired muscle development [28], [29]. Thus, we examined the muscle morphology and fiber type composition of E18.5 Wnt4−/− mutant embryos compared to control littermates. No difference in the embryos weight or in the limb mass of Wnt4−/− mutants compared to wild type was observed (data not shown). Arrangement and gross structure of muscle fibers revealed by histological analysis of limb muscle sections appeared to be unaltered in Wnt4−/− mutant embryos (Fig. 5A). Moreover, in agreement with our *in vitro* results, there was no significant difference in size (circumference) of Wnt4−/− muscle fibers compared to muscles from wild type suggesting that Wnt4 is not essential for myotube formation and fusion (Fig. 5B). We then analyzed muscle fiber type composition. Hind limb cross sections (soleus level) of stage E18.5 Wnt4−/− embryos and control littermates were stained with myosin heavy chain I (MyHCI) antibodies to reveal slow muscle fibers and the numbers of MyHCI positive fibers quantified. The lack of Wnt4 induced a two fold increase in the number of MyHCI positive cells (Fig. 5C and D). Since the muscle size is normal, this suggests that Wnt4 induces an increased ratio of slow to fast fibers.

### Wnt4 interacts with MuSK and increases MuSK level of phosphorylation

**Figure 6:**
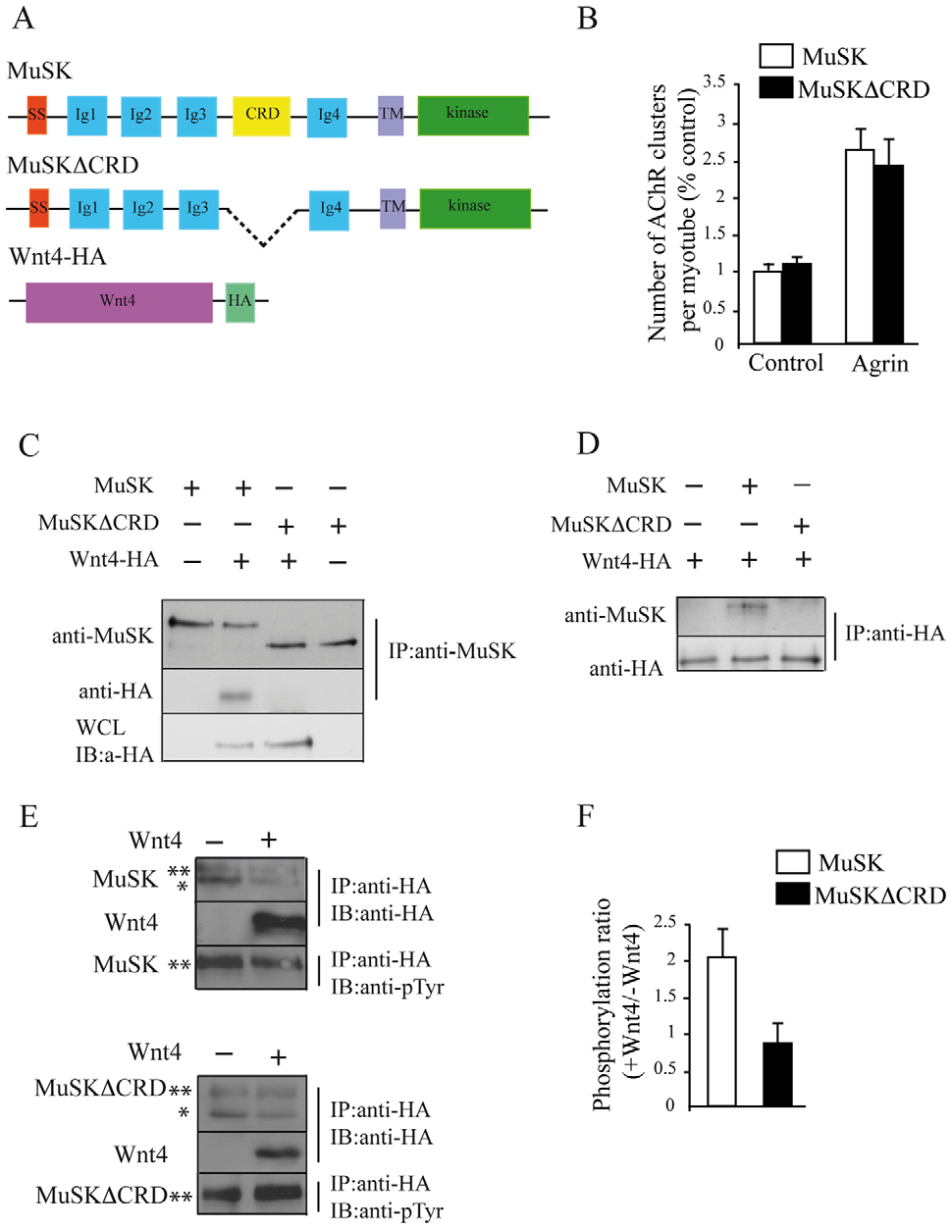
Wnt4 interacts with MuSK and increases MuSK level of phosphorylation. (**A**) Domain structure of MuSK, MuSKΔCRD and Wnt4-HA proteins. SS, signal sequence; TM, transmembrane. (**B**) Quantification of AChR cluster numbers in control or agrin treated myotubes transfected with MuSK or MuSKΔCRD. The deletion of MuSK CRD domain did not affect agrin-induced AChR clustering. (**C, D**) Coimmunoprecipitation of MuSK/Wnt4 in COS 7 cells. COS 7 cells were cotransfected with Wnt4-HA and MuSK or MuSKΔCRD. Western blot using HA antibodies was performed on cell lysates to assess the expression of Wnt4-HA (C, WCL, Whole Cell Lysate). Western blot of MuSK or HA immunoprecipitates probed with HA or MuSK antibodies showed that Wnt4 interacted with MuSK but not with MuSKΔCRD. (**E**) MuSK phosphorylation induced by Wnt4. HEK 293T cells were cotransfected with HA-MuSK or HA-MuSKΔCRD with or without Wnt4-HA. HA-MuSK, HA-MuSKΔCRD and Wnt4-HA were immunoprecipitated with HA antibodies. Western blots of HA immunoprecipitates were probed with HA or phosphotyrosine (pTyr) antibodies to assess HA-MuSK or HA-MuSKΔCRD tyrosine phosphorylation level. (**F**) Quantification of HA-MuSK or HA-MuSKΔCRD phosphorylation levels normalized to the total amount of MuSK or MuSKΔCRD proteins expressed as the +Wnt4/−Wnt4 ratio. Wnt4 induced MuSK but not MuSKΔCRD phosphorylation. Error bars show means ± SEM from three independent experiments. *: non phosphorylated MuSK, **: phosphorylated MuSK.

MuSK contains within its extracellular domain a Fz-like cysteine rich domain (CRD) homologous to the Wnt binding domain on Fz receptors (see Fig. 6A). In addition, in zebrafish Wnt11r binds *unplugged*/MuSK, prompting us to determine whether MuSK is involved in Wnt4-elicited responses [3]. First, we investigated whether MuSK can interact with Wnt4 through its CRD domain. Control experiments performed on muscle cells in culture transfected with HA-tagged MuSK or MuSK lacking the CRD domain (MuSKΔCRD) and treated with or without agrin showed that agrin-induced AChR clustering was not affected in MuSKΔCRD transfected myotubes (Fig. 6B). Moreover, MuSKΔCRD was detected at the membrane of transfected myotubes (data not shown) suggesting that the deletion of the CRD domain did not impair MuSK localization nor agrin/MuSK function on AChR clustering. MuSK or MuSKΔCRD and HA-tagged Wnt4 (Fig. 6A) were cotransfected into COS 7 cells and immunoprecipitation of the complex was performed with MuSK or HA antibodies followed by western blotting with HA or MuSK antibodies (Fig. 6C and D). Wnt4 and full length MuSK coimmunoprecipitated and this interaction was undetectable when MuSK lacks its CRD domain (Fig. 6C and D). We further asked whether the binding of Wnt4 ligand can induce MuSK activation. HEK 293T cells were cotransfected with both HA-tagged MuSK or MuSKΔCRD in the presence or absence of Wnt4-HA expression vectors and MuSK or MuSKΔCRD phosphorylation was evaluated using phosphotyrosine antibodies (Fig. 6E). Wnt4 induced a two fold increase in MuSK phosphorylation that was abolished when MuSKΔCRD domain is deleted (Fig. 6F). Together, these results indicate that Wnt4 binds MuSK through MuSK CRD domain and that this binding leads to an increase level of MuSK phosphorylation.

## Discussion

We have investigated the role of Wnt4 in mice NMJ formation *in vivo*. Our studies were prompted by the Wnt4 profile of expression during muscle development. Wnts proteins are attractive or repulsive guidance signals that regulate axon guidance and synapse assembly [14], [30]. For example, in Drosophila, Wnt4 acts as a repulsive cue controlling muscle innervation topography [31]. In contrast, in vertebrates, Wnt4 is known to act as an attractive cue involved in the anterior-posterior guidance of rat commissural axons [32]. At the mammalian NMJ, little is known about the signals regulating the early steps of nerve-muscle recognition and NMJ formation. Several lines of evidence in our study support a role for Wnt4 in these processes. First, the temporal expression pattern of Wnt4 mRNA in muscle *in vitro* and *in vivo* matches the timing of the early step of muscle innervation. Second, loss of Wnt4 function causes defects in axon pathfinding and a subset of AChR clusters are found uninnervated. Third, Wnt4 is expressed in muscle and not in motoneurons at an early stage of synapse formation. Fourth, Wnt4 binds and activates MuSK, a kinase receptor essential for pre and postsynaptic differentiation. Recently, it has been shown that the double mutant mice for Six1/4 homeoproteins have reduced level of Wnt4 expression and display a similar NMJ phenotype compared to Wnt4 null mice, further supporting a role for Wnt4 in the innervation process [33]. Thus, our data place Wnt4 in the pathway of early NMJ formation, though the fact that the Wnt4 null phenotype does not phenocopy the MuSK null phenotype suggests that they are other interactors in this process. In this line, and although so far there is no evidence in the literature supporting the expression of other Wnt receptors including Frizzled and ROR in muscle or motoneurons, one can hypothesize that Wnt4 interacts with these receptors clustered by MuSK on the muscle and/or located in motor axons. Interestingly, analysis of our microarray data during muscle differentiation in culture indicates that some Frizzled and ROR receptors are expressed in muscle (data not shown).

### Wnt4 drives pre and postsynaptic defects

Our results show that at stage E18.5, the primary nerve bundle has defasciculated and axons grow widely across the muscle, bypassing AChR aggregates resulting in 30% of AChR clusters not innervated. In addition, the surface of AChR clusters and the endplate band width is drastically increased. In contrast, the localization of synaptic markers such as AChE, MuSK and rapsyn is normal and overlaps with the localization of AChR. Accordingly, the NMJ phenotype observed in muscles from stage E18.5 Wnt4 mutant embryos is likely to be the consequence of an early defect in NMJ development as nerve terminals are about to reach their destination.

Does the absence of Wnt4 affects an anterograde signal that would shape the postsynaptic domain? In this context, it is interesting to compare the Wnt4 phenotype with that of mutants in which the presynaptic function or the transduction of the presynaptic message is impaired, for example the choline acetyltransferase (ChAT), the CDK5 [34], [35]. In these embryos, the synaptic endplate band is larger as in the Wnt4 mutant. However, AChR clusters are either smaller or identical in size compared to wild type clusters and most postsynaptic sites are contacted by nerve terminals in contrast to the Wnt4 mutant. Interestingly, the transcription factor HB9 mutant in which the phrenic nerve is absent resulting in a non innervated diaphragm displays small AChR clusters in contrast to the observed phenotype in Wnt4 deficient mice [36], [37], [38]. Thus, Wnt4 deficient synapses present specific traits that are not found in mutants carrying presynaptic defects. The defects in Wnt4 mutant innervation could be the result of a lack or delay in motoneuron or muscle differentiation. However, the group of Jessell and colleagues have shown that a redundancy between Wnt4 and Wnt5 insures that motoneurons differentiate and acquire proper connectivity [25]. This excludes the possibility that Wnt4 deficiency alone could influence muscle innervation through mechanism linked to motoneuron abnormal development. All together these observations seem at odds with the possibility that presynaptic defects alone account for the postsynaptic phenotype in absence of Wnt4 at E18.5 and argue against Wnt4 acting through an anterograde signal.

### Wnt4 is expressed by muscle cells and is enriched at the postsynaptic domain at an early stage of synapse formation

The overall structure of Wnt4 deficient muscle appears normal in terms of muscle mass, myotube formation and muscle fiber diameter. However, we found that the lack of Wnt4 induces an increase in the number of slow fibers. This is in line with previous studies showing that Wnt4 can modulate the ratio of fast to slow muscle type fibers [28], [29]. During embryonic myogenesis (E10.5 to E12.5), primary fibers mostly express slow fiber genes whereas secondary fibers appearing later during fetal myogenesis (E14.5 to E17.5) mostly express fast fiber genes [39]. Thus, one possibility is that in the Wnt4 mutant a subset of pre-programmed secondary fibers are unable to acquire a fast phenotype and retain a slow type phenotype. In this context, although mechanisms underlying synapse specificity during motor unit formation are still obscure, a subset of motoneurons may not been able to recognize their specific target fibers at the right time. However, since diaphragm and intercostal muscles present the same percentage of uninnervated postsynaptic domains (data not shown) although they have different fiber-type composition, this explanation seems unlikely.

Our data revealed that Wnt4 mRNAs are highly expressed by the muscle at the time when the first synaptic contacts are formed *in vivo* and that this expression peaks shortly after myotubes are formed *in vitro*. More, the expression of Wnt4 mRNA is enriched at the neuromuscular junction. Since Wnt4 is not expressed by the ventral zone of the developing spinal cord where motoneurons are located, a presynaptic source of Wnt4 could be provided by Schwann cells. However since Schwann cells track along axons and do not reach NMJs until axons have made contact with muscles, Schwann cells defective for Wnt4 should not affect the early steps of muscle innervation [40]. Our *in vitro* results support a role for muscle secreted Wnt4. First, Wnt4 is expressed in a cell line differentiating exclusively into muscle cells. Second, Wnt4 affects AChR clustering *in vitro* in the absence of innervation indicating that muscle cells express a Wnt4 receptor. Third, addition of Wnt4 on muscle cells *in vitro* leads to an increase of AChR clusters whereas in the absence of Wnt4, the number of prepatterned AChR is decreased. This last observation shows that Wnt4 is dispensable for the expression of prepatterned AChR but that it controls this process before muscle innervation.

### What mechanisms mediate Wnt4 function in synaptogenesis?

We report that Wnt4 binds to the extracellular CRD domain of MuSK and increases MuSK phosphorylation levels, an interaction that can be correlated to the innervation process. Our results are consistent with data showing that MuSK dramatically regulates axonal outgrowth in mice as well as in zebrafish [4], [41]. A tempting hypothesis would be that Wnt4 acts on centrally located MuSK and participates to the formation of an attractive complex for axons by a retrograde mechanism. Its absence does not prevent AChR prepatterning but modify the composition of the postsynaptic domain that is reflected by a modification of the number of AChR clusters. Since Wnt proteins can regulate MuSK expression and that the clustering of AChR is largely controlled by MuSK, an explanation for the innervation defects would be that the lack of Wnt4 induces a downregulation of MuSK expression at the stage of prepatterning [42]. This would in turn modify the expression of a retrograde signal and decrease the efficacy of the axon-muscle recognition. At later embryonic stages, compensatory mechanisms intrinsic to the muscle stimulate AChR clustering and maintain the existence of AChR clusters left unapposed by nerve terminals, a striking feature of the Wnt4 mutant. The identity of the cascade downstream of Wnt4/MuSK and the retrograde signal controlling axon targeted growth remain to be elucidated.

An interesting feature of Wnt4/MuSK interaction is that it occurs in the absence of LRP4, the agrin co-receptor for MuSK. Yet Wnt4 induces an increase level of MuSK phosphorylation. Thus, Wnt4 can elicit a MuSK specific pathway independently of the agrin-MuSK pathway. The putative existence of two independent muscle signaling pathways is supported by the fact that different phenotypes for Wnt4 and agrin mutants are observed. Indeed, agrin mutant mice have fewer sites of postsynaptic differentiation and AChR clusters are smaller and less intense, a phenotype opposite to that of the Wnt4 mutant [43].

In the Wnt4 mutant, a number of postsynaptic domains remain innervated suggesting that Wnt4 might act together with another partner sequentially or at the same time to fully coordinate nerve and muscle apposition in the early phase of NMJ formation. During the same phase in zebrafish, the requirement for additional molecules besides Wnt11r in MuSK signaling has also been reported [3]. Therefore, more factors are to be identified to recapitulate MuSK function in muscle innervation.

## Materials and Methods

### Mice embryos and Antibodies

E14 and E18.5 embryos from Wnt4 mutant mice or control littermates were obtained from A. Swain [44]. E11.5 to E18.5 wild-type mice embryos were purchased from Janvier (France). All mouse work was performed in accordance with French legislation and reviewed by the local ethical committee of the Paris Descartes University. The investigators had valid licenses to perform experiments on live vertebrates delivered by the Direction des Services Veterinaires (Prefecture de Police, Paris, France). The animal house and the experimental room of Paris Descartes University had received the agreement of the same authority (N° B75-06-07).

The following antibodies were used: Alexa Fluor® 488 conjugated (polyclonal and monoclonal, Invitrogen Molecular probe, 1/1000), Cy™ 3-conjugated (polyclonal, Jackson immunoresearch, 1/1000), Cy5™ 5-conjugated (monoclonal, Jackson Immunoresearch, 1/1000), Peroxidase conjugated light chain specific (monoclonal, Jackson immunoresearch, 1/10000), Peroxidase conjugated (monoclonal and polyclonal, Jackson immunoresearch, 1/10000), α-bungarotoxin (α-BGT) Alexa Fluor® 594 conjugate (Invitrogen Molecular Probes, 1/1000), Synaptophysin (monoclonal, Zymed, 1/5), Rapsyn (clone 1234, Sigma-Aldrich, 1/40), Neurofilament (polyclonal, Chemicon, 1/2000), HA.11 (clone 16B12 and polyclonal, Eurogentec, 1/1000), HA (polyclonal, Sigma, 1/1000), Phosphotyrosine (clone 4G10, Millipore, 1/500), Myosin heavy chain 1 (MyHCI, monoclonal, Sigma, 1/1000) and MuSK (polyclonal, Abcam, 1/200). A63 is a rabbit polyclonal anti-AChE raised against rat AChE [45]. Rabbit polyclonal anti-MuSK antibody (1/500) used for immunohistochemistry is a gift from M. Ruegg (Germany).

### COS 7, HEK 293T and Muscle cells culture

The wild type muscle cell line was generated from one week-old mice as described in Cartaud et al. (2004). Myoblast cells were cultured on plates coated with collagen Type I (Iwaki, Japan) maintained in DMEM supplemented with 10% fetal bovine serum, 20% horse serum, 2 mM glutamine, 2% penicillin/streptomycin (5,000 U) and 20 U/ml of γ-interferon (Roche Diagnostics; Mannheim, Germany) at 33°C in 8% CO_2_. All the culture medium reagents were purchased from Invitrogen. Cells were differentiated into myotubes in the same medium containing 5% horse serum without γ-interferon (differentiation medium). Three stages of muscle cell differentiation were selected for analysis: T1 when cells are mostly myotubes (day 0), T2 (day 2) when AChR clusters are visualized and T3 (day 5) when both AChR and AChE clusters are observed [23]. When indicated, recombinant Wnt4 protein (R&D system) was added overnight to stage T2 myotubes at a final concentration of 10 ng/ml.

COS 7 cells and 293T cells (ATCC) were cultured in DMEM supplemented with 10% fetal bovine serum, 2 mM glutamine and 2%penicillin/streptomycin (500 U) at 37°C in 5% C0_2_.

### In Situ Hybridization

Spinal cord frozen sections (thoracic level) from E115 and E13.5 wild type embryos were fixed in 4% formaldehyde, digested with proteinase K, hybridized with digoxigenin-labeled riboprobes directed against the mRNA encoding *Wnt4* (GenBank accession number NM_009523.1) and processed as described previously [2]. Labeling with sense probes resulted in weak, uniform staining (data not shown).

### Microarray analysis and SYBR Green RT-PCR

Total hind limb RNAs from various developmental stages embryos and newborn mice, total RNAs from muscle cells in culture (T1, T2 and T3 time points) as well as total RNAs from synaptic and extrasynaptic regions of E18.5 diaphragms were extracted as previously described [23].

To obtain synaptic versus extrasynaptic regions, E18.5 wild type diaphragms were stained with Alexa-488-conjugated-α-bungarotoxin (α-BGT, 10 µg/ml) and synapse “rich” and extrasynaptic regions were dissected as previously described [46].

For microarray analysis, RNA from muscle cells were compared at T1, T2 and T3 (N = 3 for each time point) using Applied Biosystems Mouse Genome Survey Microarrays, containing probes representing approximately 32.000 mouse genes from the public and Celera databases. 500 ng of total RNA was reversed transcribed generating a first strand cDNA then converted in cRNA (NanoAmp RT-IVT labeling Kit) and hybridized to the mouse microarray following the manufacturer's instructions. Chemiluminescence detection, image acquisition and analysis were performed using Applied Biosystems Chemiluminescence Detection Kit and Applied Biosystems 1700 Chemiluminescent Microarray Analyzer. The set of probes differentially expressed between T1, T2 and T3 time points were determined by Student test.

SyBR Green RT-PCR experiments were performed as described previously [23]. *Wnt4* and *MuSK* QuantiTect primers were purchased from Qiagen.

### Plasmids

The rat MuSK cDNA clone and MuSK-HA have been previously described [47]. HA tag was introduced at the NH2 terminus. MuSKΔCRD or MuSKΔCRD-HA expression vectors have been constructed by PCR deletion of the corresponding CRD amino acids sequence [48]. The Wnt4-HA construct was a gift from Bacou F. (UMR 866, France). The sequence of the tag HA was introduced just before the stop codon.

### Transfection and Immunoprecipitation

Cells were grown to 70% confluence, transfected (2 to 7 µg of plasmids) using Fugen or Calcium phosphate transfection techniques and protein extraction was performed 36 hours later. MuSK and HA immunopecipitation were performed on total cell lysates incubated with 4 µg of anti-MuSK or anti-HA antibodies. Proteins were precipitated with protein A or G sepharose (3 hours at 4°C) according to the manufacter's instructions (GE Healthcare). Immunoprecipitates were then washed in lysis buffer (Tris HCl 50 mM, NaCL 150 mM, EDTA 2 mM, SDS 0.1%, TritonX100 1%), solubilized in SDS sample buffer and run on a 8% SDS-PAGE using a Bio-Rad Laboratories Mini-Protean III slab cell. Proteins separated by gel electrophoresis were then transferred to nitrocellulose membrane (Schleicher and Schuell). Western blot using anti-MuSK, anti-HA or anti-phosphotyrosine antibodies were performed as described previously [49], revealed with enhanced chemiluminescent detection (ECL+; Amersham Pharmacia Biotech), and exposed to Fuji X-ray films. Relative signal intensity of total and phosphorylated MuSK or MuSKΔCRD proteins was measured using ImageJ software and tyrosine phosphorylation levels were normalized to the total amount of MuSK or MuSKΔCRD proteins.

### Immunohistochemical analyses

For whole mount analyses, dissected intercostal or diaphragm muscles from E13.5 and E18.5 control littermate or Wnt4−/− embryos were fixed (4% formaldehyde in phosphate buffered saline) (PBS) for 90 minutes at room temperature and further fixed (1% formaldehyde in PBS) overnight at 4°C. Muscles were washed three times for 15 minutes in PBS, incubated for 15 minutes with 100 mM glycine in PBS and rinsed in PBS. Muscles were permeabilized (0.5% Triton X-100 in PBS) for 10 minutes and blocked for 1 hour in DakoCytomation Protein block (Glostrup, Denmark). Axons and nerve terminals were labeled by staining muscles overnight at 4°C with rabbit polyclonal antibodies against neurofilament (NF) and synaptophysin (SYN) in blocking solution. After three 1-hour washes in PBS, muscles were incubated 3 hours with Alexa-488 goat anti-rabbit IgG and Alexa-594-conjugated-α-bungarotoxin (α-BGT) in blocking solution, to label AChRs. After three 1-hour washes in PBS, muscles were post-fixed (1% formaldehyde in PBS) for 10 minutes, rinsed in PBS and mounted under glass in Vectashield (Vector Labs, Burlingame, CA).

For sections analyses, dissected hind limb muscles from E18.5 or E14 control littermate or Wnt4−/− embryos were fixed (1% paraformaldehyde in PBS) for 1 hour at 4°C, rinsed twice at 4°C in PBS, cryoprotected (30% sucrose-PBS) overnight at 4°C, and embedded in TissueTek (Sakura, Tokyo, Japan). Cross sections (12 µm) were labeled with various antibodies as the same as for whole mount immunostaining.

For histological analysis, muscle sections were stained with heamatoxylin/Eosin according to the manufactor's instruction (Sigma-Aldrich, France).

For muscle cross section perimeter analysis, hind limb muscle sections were stained with lectin-Fitc overnight at 4°C. Muscle sections perimeter were then quantified using ImageJ software.

For myotube staining, muscle cells were fixed (4% paraformaldehyde in PBS) for 1 hour, at room temperature, permeabilized (0.5% Triton X-100 in PBS) and AChR clusters were stained with α-BGT for 1 hour at room temperature. Cells were subsequently washed and mounted in Vectashield.

### Image acquisition and Processing

All images were collected on a microscope (model BX61; Olympus) equipped with a Fast 1394 Digital CCD FireWire camera (model Retiga 2000R; Qimaging) and a 40× oil objective (numerical aperture: 1.0; Olympus) or on a confocal laser scanning microscope (Zeiss LSM-510) equipped with a 20× and a 40× oil objective. Collected Z-stacks confocal images (5 to 16 stacks with 1 (20×) or 0.5 µm (40×) z-steps) and image capture were made using LSM Image Browser or ImageProPlus softwares (version 5.1). The same laser power and parameter setting were applied to ensure fair comparison between wild type and Wnt4−/− embryos. Confocal images presented are single-projected image derived from overlaying each set of stacks.

For quantification of the individual AChR clusters surface and intensity, image stacks were quantified using the ImageJ (version 1.37c) plugin “3D object counter” [50]. The threshold intensity was set by visual inspection of AChR clusters (40). The AChR endplate band width was measured by drawing a polygon connecting the most peripheral AChR clusters and calculating the average myotube length contained in the polygon using ImageJ software [35].

### Statistical Analysis

Data were expressed as means ± SEM. Statistical analyses were performed with Graphpad InStat3 using the Mann-Whitney U-test (*P*<0.05 considered significant). Each experiment was conducted a minimum of three times.
